# The Evolutionary Kaleidoscope of Rhodopsins

**DOI:** 10.1128/msystems.00405-22

**Published:** 2022-09-19

**Authors:** Paul-Adrian Bulzu, Vinicius S. Kavagutti, Adrian-Stefan Andrei, Rohit Ghai

**Affiliations:** a Biology Centre of the Czech Academy of Sciences (CAS), Institute of Hydrobiology, České Budějovice, Czech Republic; b Department of Ecosystem Biology, Faculty of Science, University of South Bohemia, České Budějovice, Czech Republic; c University of Zurich, Limnological Station, Microbial Evogenomics Lab (MiEL), Kilchberg, Switzerland; Northwestern University

**Keywords:** rhodopsins, Alt-rhodopsins, AltRs, heliorhodopsins, optogenetics, metagenomics

## Abstract

Rhodopsins are widely distributed across all domains of life where they perform a plethora of functions through the conversion of electromagnetic radiation into physicochemical signals. As a result of an extensive survey of available genomic and metagenomic sequencing data, we reported the existence of novel clades and exotic sequence motifs scattered throughout the evolutionary radiations of both Type-1 and Type-3 rhodopsins that will likely enlarge the optogenetics toolbox. We expanded the typical rhodopsin blueprint by showing that a highly conserved and functionally important arginine residue (i.e., Arg82) was substituted multiple times during evolution by an extensive amino acid spectrum. We proposed the umbrella term Alt-rhodopsins (AltRs) for all such proteins that departed Arg82 orthodoxy. Some AltRs formed novel clades in the rhodopsin phylogeny and were found in giant viruses. Some newly uncovered AltRs were phylogenetically close to heliorhodopsins, which allowed a closer examination of the phylogenetic border between Type-1 rhodopsins and heliorhodopsins. Comprehensive phylogenetic trees and ancestral sequence reconstructions allowed us to advance the hypothesis that proto-heliorhodopsins were a eukaryotic innovation before their subsequent diversification into the extant Type-3 rhodopsins.

**IMPORTANCE** The rhodopsin scaffold is remarkably versatile and widespread, coupling light availability to energy production and other light-dependent cellular responses with minor alterations to critical residues. We described an unprecedented spectrum of substitutions at one of the most conserved amino acids in the rhodopsin fold, Arg82. We denoted such phylogenetically diverse rhodopsins with the umbrella name Alt-rhodopsins (AltR) and described a distinct branch of AltRs in giant viruses. Intriguingly, some AltRs were the closest phylogenetic neighbors to Heliorhodopsins (HeRs) whose origins have remained enigmatic. Our analyses of HeR origins in the light of AltRs led us to posit a most unusual evolutionary trajectory that suggested a eukaryotic origin for HeRs before their diversification in prokaryotes.

## INTRODUCTION

Rhodopsins are remarkably promising molecules for modulating cell expression with precision (1–3), but for many their biological role in the natural environment remains largely obscure. With increasing sequence data, more rhodopsins are being found (4–9), but it is unclear to what extent the sequence diversity of the rhodopsin-verse has been explored. Type-1 (microbial rhodopsins) and Type-2 (animal rhodopsins) share similar, seven-helical topological conformation and membrane orientation with the N terminus in the extracellular space and a Schiff base linkage from a conserved lysine to retinal in the seventh helix (TM7) (10). However, while the overall fold is the same, there is no detectable sequence similarity between these two types. A completely new type of rhodopsin similar to Type-1 rhodopsins was identified recently but with inverse membrane orientation (Heliorhodopsins, HeRs, or Type-3 rhodopsins) (11). Despite their orientation, HeRs also bind the retina using a conserved lysine in TM7 (transmembrane helix 7). Apart from the lysine in TM7 that is essential for binding retinal, several other functionally important residues have been identified, e.g., several characteristic sequence motifs in TM3 and TM7 that may be predictive of the nature of the ion pump. Proteorhodopsins typically display DTE or DTD motifs in TM3 and a DxxxK motif in TM7. Inward chloride pumps may be recognized by NTQ or TSD motifs in TM3 and a DxxxK motif in TM7 and heliorhodopsins have the ESL motif in TM3 and SxxxK in TM7 (12).

One critical and highly conserved residue is Arg82 (BR numbering) in the third transmembrane helix (TM3). Since the discovery of bacteriorhodopsin (BR) in haloarchaea nearly 5 decades ago (13), no naturally occurring rhodopsins are known that do not have a conserved Arg82 residue (12). Among conserved BR amino acids, Arg82 in TM3 was recognized as an essential player within the photocycle due to its involvement in proton release through interactions with Asp85, Asp212, and the retinal Schiff base (14, 15). Such conclusions are the result of experimental work and observations from multiple mutagenesis studies that describe the effects of targeted Arg82 substitutions on BR photocycle and proton release: R82A (14, 16–18), R82C (16), R82H (15), R82K (18–20), and R82Q (14, 17, 20, 21). In general, these studies indicate that the charge and hydrogen bonding capabilities of the residue in position 82 drastically influence the interactions between the proton acceptor (Asp85) and proton release group, thus altering or even abolishing proton release to the extracellular space under normal physiological conditions (14–16, 18). This changed with the discovery of xenorhodopsins (22) that were shown to have a tryptophan (W) or phenylalanine (F) in this position and to function as unusual inward proton pumps (23). Since then, a few substitutions to Arg82 were reported in (i) anion channels (K instead of R) (4), (ii) a few rhodopsins of unknown function (Q, A, and T instead of R) (6), and (iii) potassium pumps (kalium rhodopsins, W instead of R) (5). However, no such substitutions have ever been described in HeRs.

In this work, we performed an extensive search through hundreds of metagenomes and metatranscriptomes and showed that a surprisingly large number of rhodopsins with peculiar amino acid substitutions and novel motifs had remained out of sight. We utilized this newly unearthed diversity to construct large-scale, highly supported phylogenies and to generate a plausible evolutionary scenario for the origin and evolution of Type-3 rhodopsins (heliorhodopsins; HeRs) (11).

## RESULTS AND DISCUSSION

### Novel clades of unusual rhodopsins.

We scanned large collections of genomic, metagenomic, and metatranscriptomic data sets from various sources (e.g., marine, freshwater, brackish, sediments, prokaryotic/eukaryotic genomes, and eukaryotic transcriptomes) to identify novel rhodopsin sequence variants. Sequences with seven transmembrane helices and a conserved lysine in TM7 were considered bonafide rhodopsins (see Materials and Methods for details). We aligned these sequences with known rhodopsins to identify characteristic motifs in TM3 and TM7. Unexpectedly, these alignments also revealed substantial variation in residue 82 (Arg82) that had not been observed before. We found that the known substitutions for this critical TM3 residue (i.e., Arg82) could be significantly expanded by additional changes at this position in both Type-1 and Type-3 rhodopsins. In sum, we found strong evidence supported by at least 10 sequences in each case that residues H, K, Q, A, P, S, Y, E, and M can replace Arg82. Other substitutions (N, I, F, T, L, G, D, V, and C) were also identified but less than 10 times (see Table S1). We proposed the umbrella name Alt-rhodopsins (AltRs) for all such microbial rhodopsins with a substituted Arg82 (i.e., non-R type rhodopsins as opposed to R-type ones with Arg82). This nomenclature included xenorhodopsins and kalium rhodopsins as subtypes of Alt-rhodopsins. Here, we differentiated among the subtypes of Alt-rhodopsins by indicating the substituent amino acid symbol (e.g., H-type when H replaced the canonical R).

10.1128/msystems.00405-22.2 TABLE S1 Summary of all rhodopsins included within this study. Download Table S1, XLSX file, 0.4 MB. Copyright © 2022 Bulzu et al. 2022 Bulzu et al. https://creativecommons.org/licenses/by/4.0/ This content is distributed under the terms of the Creative Commons Attribution 4.0 International license.

**FIG 1:**
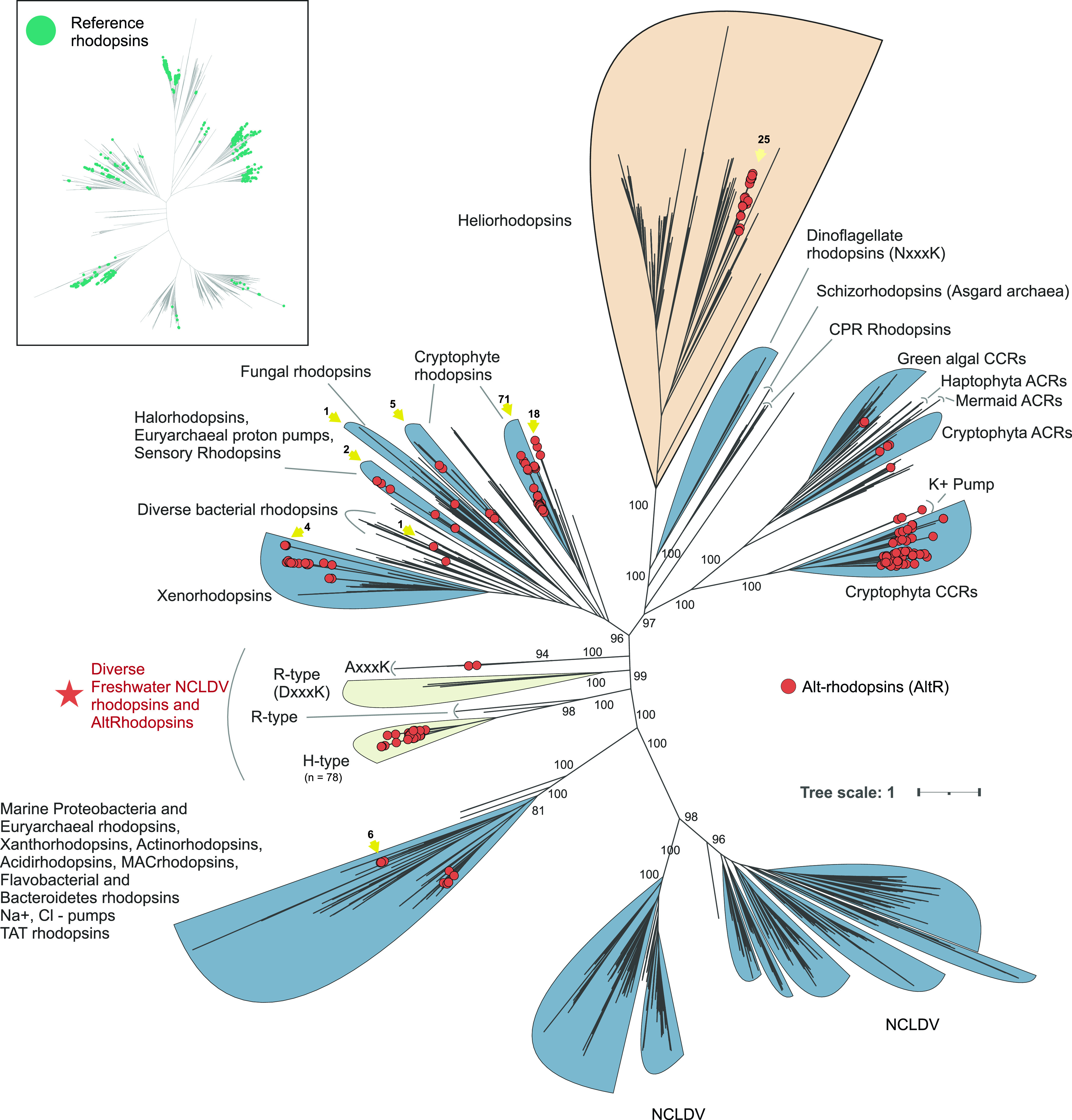
Maximum likelihood phylogenetic tree of rhodopsins. Alt-rhodopsins are indicated by red circles at node tips. Ultrafast bootstrap values are shown at selected nodes. A star indicates the novel clades of rhodopsins and Alt-rhodopsins. Yellow arrows indicate the position of H-type rhodopsins and their counts. The inset at the top left shows a simplified version of the phylogenetic tree marking all reference rhodopsin sequences (in green circles). See also Tables S1 and S2 and FigShare Data at https://figshare.com/s/f2d7b1065930bf350c2f.

AltRs were present in nearly all phylogenetic clades of rhodopsins in bacteria, archaea, eukaryotes, and viruses (Fig. 1) (insofar as taxonomic origin could be reliably ascribed). However, by far the vast majority (*n* = 102) were found in the eukaryotic Cryptophytes. Altogether, we classified 399 sequences as AltRs (see Table S2). At least 121 of these sequences originate from previously described channelrhodopsins found in unicellular algae and giant viruses (4), xenorhodopsins (22), and potassium pumps (5), all classified as Type-1 rhodopsins. The previously undescribed sequences (*n* = 278) encompassed both Type-1 and HeRs with 93 of them being of confident taxonomic origin, with 30 from eukaryotes (26 cryptophytes, 1 fungus, 1 ciliate, and 2 unclassified), 34 of nucleocytoplasmic large DNA viruses (NCLDVs), 25 bacterial (12 proteobacteria, 9 actinobacteria, 3 cyanobacteria, and 1 Verrucomicrobiota) and 4 archaeal (3 Halobacteriota and 1 Thermoplasmatota). Of the sequences with uncertain taxonomy (*n* = 185), 110 were likely eukaryotic, 42 were bacterial, and the remaining 33 were unclassified. This distribution implied that AltRs were universally distributed across all domains of life and appeared to be present in both monoderm and diderm bacteria. This contrasted with heliorhodopsins, which are restricted to monoderms (9, 24).

10.1128/msystems.00405-22.3 TABLE S2 Summary of Alt-rhodopsins. Download Table S2, XLSX file, 0.1 MB. Copyright © 2022 Bulzu et al. 2022 Bulzu et al. https://creativecommons.org/licenses/by/4.0/ This content is distributed under the terms of the Creative Commons Attribution 4.0 International license.

### Giant viruses encoded H-type Alt-rhodopsins.

Multiple, phylogenetically distinct lineages of rhodopsins (including HeRs) revealed signs of widespread convergent evolution, which was evident from the dispersed distribution of non-R type rhodopsins. For example, H-type AltRs (*n* = 214), which were the most common novel type, did not form a phylogenetically coherent lineage but seemed to have emerged independently in multiple lineages (indicated with yellow arrows in Fig. 1). Additionally, we observed multiple closely related clades of Type-1 rhodopsins accommodating both classical rhodopsins (with Arg82) and H-type AltRs (Fig. 1, indicated by a star). Clustering of AltR encoding contigs and transcript (wherever available), based on shared gene content, also revealed the same major group (*n* = 47, indicated by a star in Fig. 1). However, their taxonomic origin remained unclear. By analyzing flanking genes near these H-type rhodopsins within these contigs, we could identify typically eukaryotic genes, such as the mRNA capping enzyme (mRNAc) and DNA-dependent RNA polymerase subunits (RNAPL) (See Fig. S1). Such genes, however, are also encoded by giant viruses (25). Further scanning of these contigs using ViralRecall (26) convincingly identified them as belonging to the broad class of nucleocytoplasmic large DNA viruses (NCLDVs) with all contigs showing at least one positive hit to known viral proteins. Among contigs ≥5 kb (*n* = 34) a total of 25 encoded at least one hallmark NCLDV marker gene with the longest contig (L969, ~116 Kbp) harboring 5 distinct markers (see also Tables S1 and S2). All AltRs in this cluster had the Arg82 replaced by histidine (H-Type) and display a DxxxK motif in TM7. A more detailed view of gene context variability in the vicinity (5 kbp upstream and downstream) of selected NCLDV H-type AltRs is provided in Fig. S1.

10.1128/msystems.00405-22.1 FIG S1 Gene context analysis in the vicinity of rhodopsins encoded by giant viruses. Rhodopsin genes are indicated by red-bold labels. Identical and near identical contigs were collapsed (counts indicated in brackets; total contigs *n* = 47). All rhodopsins were of type-1-H and originated from NCLDVs. For details see also Table S1 and Fig. 1. Download FIG S1, EPS file, 2.3 MB. Copyright © 2022 Bulzu et al. 2022 Bulzu et al. https://creativecommons.org/licenses/by/4.0/ This content is distributed under the terms of the Creative Commons Attribution 4.0 International license.

### The phylogenetic border between Type-1 and Type-3 rhodopsins.

The phylogenetic tree presented in Fig. 1 provided a tantalizing glimpse into the evolution of rhodopsins at large. We sought to examine the sequences closest related to HeRs in more detail to shed light on their presently mysterious evolutionary history as recent works with large numbers of rhodopsin sequences have either considered them as outgroups (27), had insufficient support for the HeR clade (1) or excluded them altogether (6).

Several clades in the tree display high statistical support suggesting they were well-resolved (Fig. 1, *n* = 2199 sequences). Moreover, it also appeared clear that Type-1 rhodopsin diversity far exceeded HeR diversity because HeRs were restricted to a single, albeit highly coherent clade. The topology also indicated that HeRs were a recent innovation and that Type-1 rhodopsins were ancestral. Additionally, HeRs appear to currently be most closely related to two distinct clades of Type-1 rhodopsins (R-type) originating from eukaryotes, one from Dinoflagellates (TM3 motifs ETK, ETS, ETC, or unusual TM7 motif NxxxK) and the other from Colpodellida, (Chromera velia, unusual TM3 motif QTQ, TM7 motif DxxxK), both Alveolates. Notably, several HeRs also shared TM3 motifs similar to the ones present in dinoflagellate rhodopsins, e.g., ESV, ETI, or ESL. The dinoflagellate sequences have been described before (4) and characterized as weak pumps of unknown selectivity (6). Their relatedness to HeRs, however, had not been reported. The next closest clade to these are inward pumping Schizorhodopsins (SzRs) found mainly in Asgard archaea and CPR bacteria (8, 28, 29).

**FIG 2:**
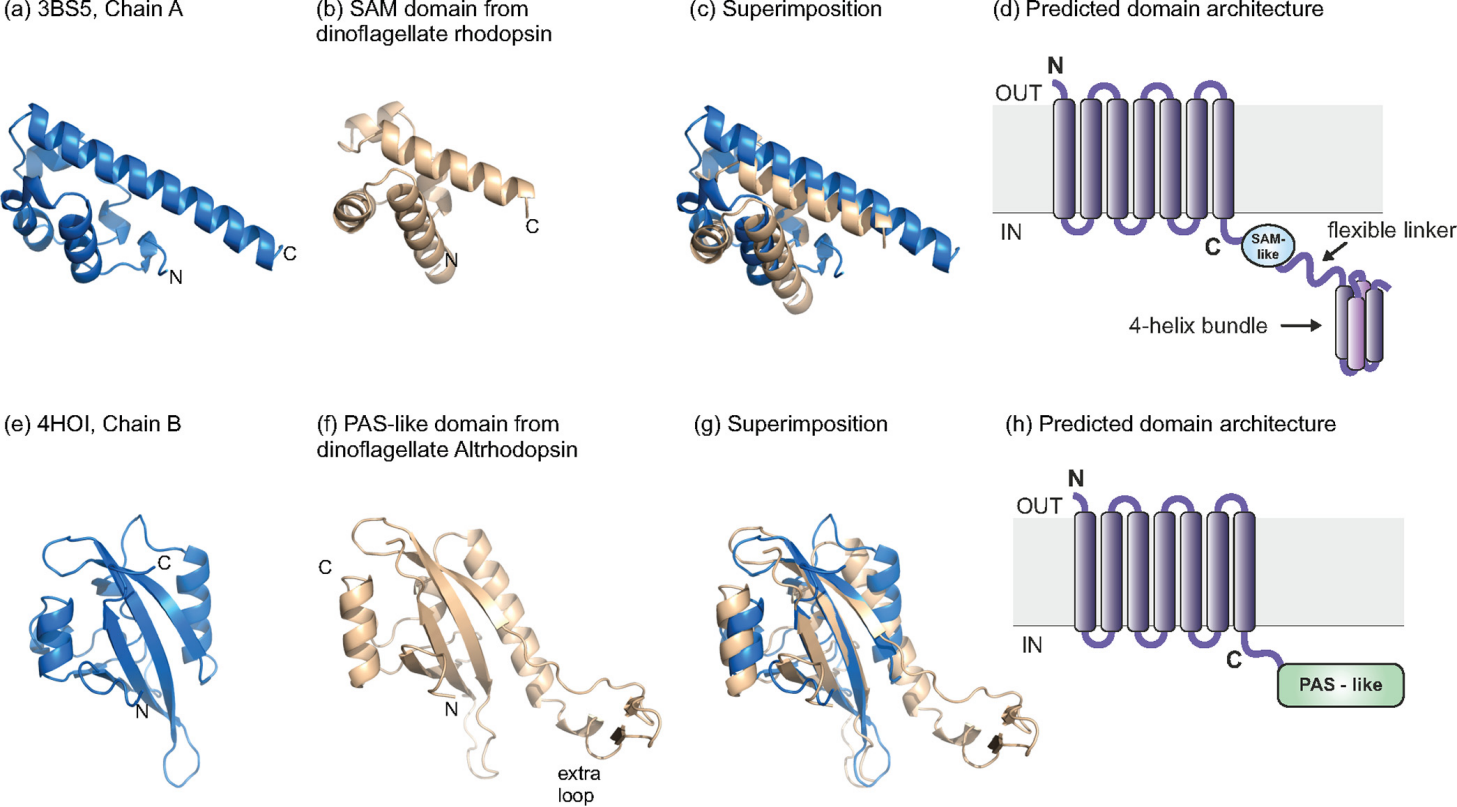
Comparison of known and predicted structural domains in C-terminal of selected dinoflagellate Type-1 rhodopsins. (A) Reference structure of a SAM domain, PDB accession no. 3BS5, (B) predicted structure of SAM domain from dinoflagellate rhodopsin sequence NCBI accession no. CAE6957589 (c) superimposition of (A and B), and (D) Predicted domain architecture of the entire protein (E) reference structure of a PAS domain, PDB accession no. 4HOI, (F) predicted structure of the PAS domain from the dinoflagellate Alt-rhodopsin (NCBI accession no. CAE7343182), (G) superimposition of (D and E), and (H) predicted domain architecture of the entire protein.

Upon closer examination, some of these unusual dinoflagellate rhodopsins (e.g., NCBI accession no. CAE6957589.1; Type-1, R-type, TM3 motif ETK, and TM7 motif NxxxK) appeared to have an extremely long C-terminal region (~500 aa) that returned no clear hits to any known sequence domain (except the N-terminal rhodopsin domain). We performed multiple iterations of structural modeling for this sequence (both entire and in multiple parts) to identify putative domains using Alphafold2 (30). The modeling results indicated that the C terminus (intracellular) of the rhodopsin domain was connected to a compact helical domain (modeled with high IDDT scores by AlphaFold2). To identify similar structures, we used this predicted model as a query for structure based-searches with VAST (31). This search identified similar structural motifs in archaeal elongation initiation factor 2 (PDB accession no. 3CW2, domain 2 in alpha subunit) (32), SAM domains (sterile alpha motif) in Yan and Mae proteins that are known to dimerize (33), and SAM domains in proteins CNK and HYP that are known to dimerize as well (PDB accession no. 3BS5) (34). Additionally, distal to the SAM-like domain, there was a flexible linker region (modeled with low support) followed by a second domain composed of multiple helical segments, which also had low modeling support but distant similarities to four-alpha helix bundle domains. The SAM-like domain, the linker, and the four-alpha helix bundle domain were all located in the cytoplasm with no transmembrane helices predicted in this domain (see Fig. 2). The SAM-like domain may likely be useful in the dimerization of such rhodopsins (35) and along with the downstream domains facilitate the transmission of the conformational change in the rhodopsin domain to initiate a signaling cascade. Several dinoflagellate rhodopsins, which showed no or low photocurrents (6) and were coupled to additional domains in the cytoplasm (like the one shown here), were indeed evocative of heliorhodopsins that showed no transport activity and may be coupled to additional domains themselves (9).

**FIG 3:**
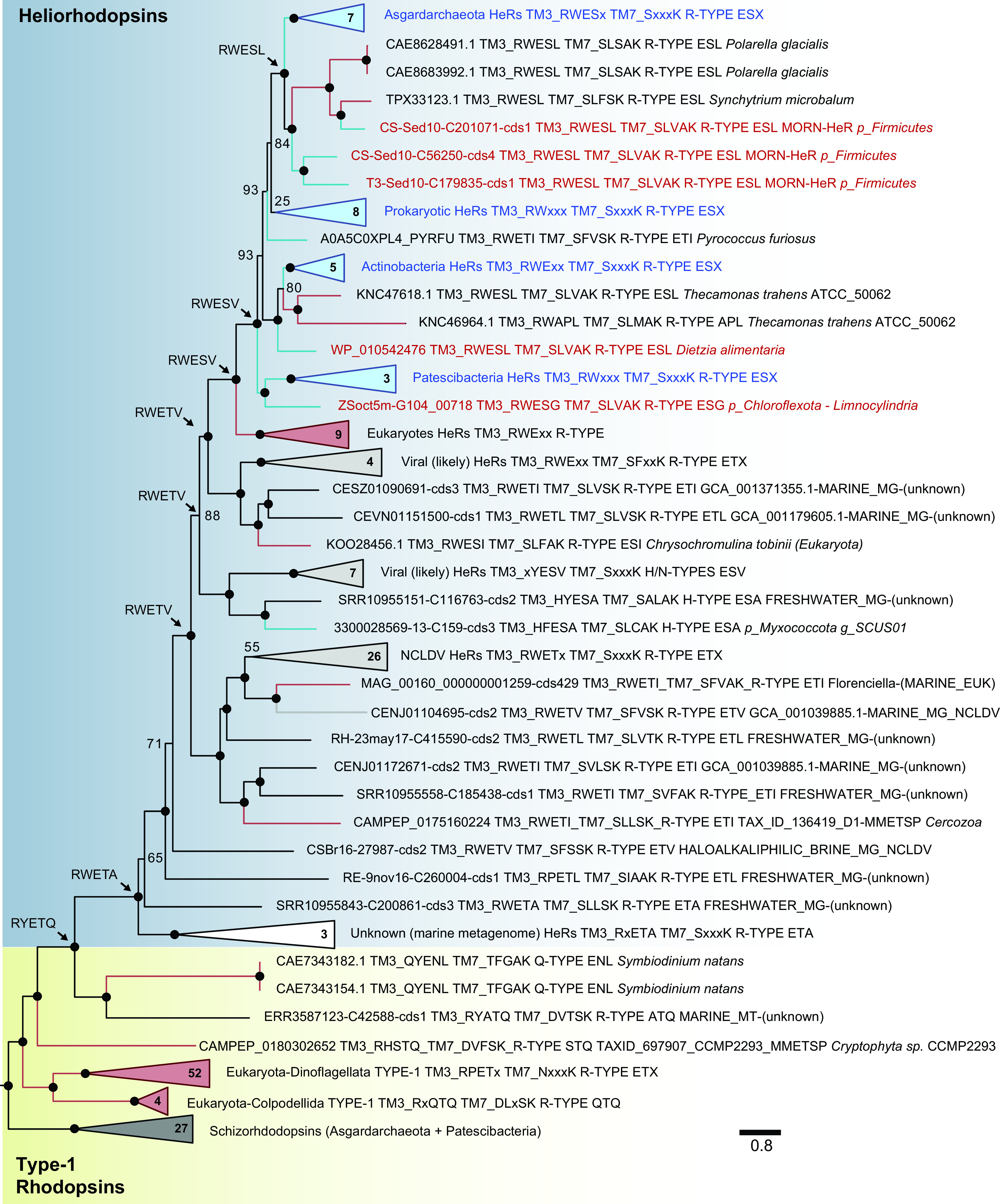
Phylogenetic tree depicting Type-1 rhodopsins and Heliorhodopsin evolutionary relationships. Black dots on branches indicate ultrafast bootstrap values between 95 and 100. Numbers of sequences are specified on triangles indicating collapsed clades. Labels of uncollapsed sequences indicate (i) sequence ID, (ii) conserved motifs found within the TM3 region of the rhodopsin, (iii) conserved motifs found within the TM7 region, (iv) type of rhodopsin as defined in Table S1, (v) conserved last 3 amino acids of the TM3 motif, and (vi) taxonomy and/or source of sequence if known. Red, reference sequences, all others are queries; red branches and triangles, eukaryotes; blue, prokaryotes; light gray, sequences retrieved from viruses (or likely viral origin); black lines and empty triangles, sequences of unknown taxonomy; dark-gray triangle, outgroup (Schizorhodopsins). Relevant TM3 motifs generated by ancestral sequences reconstruction are indicated using arrows. MG and MT were used as abbreviations for metagenome and metatranscriptome.

To examine the relatedness between heliorhodopsins and dinoflagellate rhodopsins in greater detail, we expanded our search to include additional eukaryotic genomes from dinoflagellates, fungi, etc (from Ensembl and NCBI). Thus, we reconstructed the phylogenetic tree using a subset of the initial sequences while also, including additional sequences closely related to HeRs (Fig. 3). Remarkably, our expanded search led us to identify another unusual Type-1 AltR from the dinoflagellate Symbiodinium natans (Q-type, TM3 motif QNL, and unusual TM7 motif TxxxK) that stood out as the phylogenetically closest Type-1 rhodopsin to Type-3 HeRs. The TxxxK motif in TM7 presented by this sequence was also reminiscent of the SxxxK motif found in HeRs. This sequence (NCBI accession no. CAE7343182.1) was 532 aa long and contained a rhodopsin domain in the first half of the protein. However, the other part of the protein contained no recognizable domains. We further modeled this C-terminal part using AlphaFold2 and obtained a predicted structural model with high iDDT scores (>80). Structure-based searches using VAST suggested close similarities to the PAS domain (See Fig. 2). Additional structure comparisons by TM-align (36) confirmed the same fold (TM-score >0.5; root mean square deviation (rmsd), 3.22). Notably, the PAS domain from this AltR presented an additional loop (Fig. 2). PAS domains are known to be associated with sensory proteins (37), and in several cases, may bind a wide variety of ligands, e.g., heme, hydroxycinnamic acid (38). The PAS domain fold was also found in the LOV domain that was known to bind the flavin mononucleotide (FMN) chromophore acting as a blue light sensor. Remarkably, the VAST search we performed also detected structural similarities to a LOV domain (PDB accession no. 3SW2) (39). The ligand, (if any) that would bind to this PAS domain was unclear but the close similarities to sensory LOV domains reiterate the possible sensory activity of this dinoflagellate rhodopsin. Both rhodopsin sequences that stand as phylogenetic neighbors contained motifs similar to HeRs along with additional domains possibly involved in signaling (or as yet unknown activity), which was consistent with the inference that channeling ions was perhaps not their function.

### A eukaryotic origin for heliorhodopsins.

We recently argued, based on phylogenetic evidence, that Type-1 rhodopsins were likely the more ancient rhodopsins and HeRs were a comparatively recent innovation (9). Additionally, ancestral reconstruction of Type-1 rhodopsin sequences has suggested that DTE proton pumps most likely represent the ancestral form of Type-1 rhodopsins (27). We performed ancestral reconstruction at multiple nodes in the HeR phylogeny that singled out the ETx motif as ancestral to all HeRs (see Materials and Methods for details). Additional similar ancestral motifs for different clades of HeRs were indicated in Fig. 3. The overall close phylogenetic relatedness and the similarities in the motifs (ESx, ETx) led us to posit that HeRs likely originated from such eukaryotic rhodopsins and were subsequently captured by giant viruses as well. The acquisition of HeRs by prokaryotes and their subsequent diversification in monoderms appeared to have been a later event. This unusual evolutionary trajectory of HeRs (prokaryote to eukaryote being the more common direction) (40), coupled with their fusions/co-occurrences with multiple sensory domains also suggested that HeR was cast in an enabling role as a flexible scaffold allowing innovation in cellular signaling in response to light.

With the diverse array of new rhodopsin sequences and more intermediate sequences sampled, it has become possible to tease out real evolutionary relationships. The diversity of AltRs reiterated the enormous plasticity in the rhodopsin scaffold that continued to surprise us even nearly 5 decades after its discovery (13). Specific activities of such new sequences must be examined individually as even with many structures the entire set of axioms that govern activity remain out of bounds. Even if the activity is understood, the functional role in the organism is unclear. The recent advent of improved methods for protein structure prediction is expected to boost hypothesis generation and structure-aided design. However, given the sheer diversity of both rhodopsins and the organisms that express them, the general lack of molecular tools outside classical model organisms, an enormous effort will still be required in the development of specific assays to finally cut the gordian knot of function for most rhodopsins.

## MATERIALS AND METHODS

### Sequence data and initial analyses.

We used a comprehensive collection of publicly available sequences, including the entire Genome Taxonomy Database (GTDB) (Release 95) (41), Uniprot (42), and ca. 50K Genomes from Earth’s Microbiomes (GEM) catalog (43). We also used publicly available metagenomic data from diverse data sets from all over the world, e.g., freshwater metagenomes and metatranscriptomes from multiple European freshwater sites like Rimov Reservoir, Jiricka Pond, Lake Zurich, Lake Constance, Lake Thun (9, 44–47), Lake Tanganyika in Tanzania (48), Lake Baikal in Russia (49), Amadorio and Tous Reservoirs in Spain (50, 51), Lake Mendota in the USA (52), Amazon River (53) in Brazil, the brackish Caspian Sea (54), marine metagenomic data from GEOTRACES (55), metagenomes and metatranscriptomes from TARA Oceans Expeditions (56), metagenomes from brackish sediments (8), metagenomes and metatranscriptomes from haloalkaliphilic brine and sediments (57–59), and eukaryotic culture transcriptomes from the MMETSP database (60). Metagenomic/metatranscriptomic sequences were downloaded and processed with BBMap tools available from https://github.com/BioInfoTools/BBMap/. Briefly, the bbduk.sh script from the BBmap project was used to remove low-quality reads (qtrim = rl trimq = 18), phiX and p-Fosil2 control reads as well as Illumina adapters (k= 21 ref=adapterfile ordered cardinality). Cleaned reads were assembled *de novo* with MEGAHIT v1.2.9 (61) using default parameters with a custom k-mer list: 29, 49, 69, 89, 109, 119, 129, and 149. All sequences in this work were named or retained existing names that allowed tracing them to their original data sets. We also collected reference rhodopsin sequences from a wide variety of previously published sources (1, 4, 5, 8, 22, 62–65).

### Rhodopsin identification.

Gene prediction on assembled data sets was performed using Prodigal v2.6.3 (66). Candidate rhodopsin sequences were scanned using hmmsearch (67) against existing PFAM models for Type-1 rhodopsins (PF01036), heliorhodopsins (PF18761), and a new HMM built from an alignment of known Type-1 and Type-3 rhodopsins (see FigShare Data at https://figshare.com/s/f2d7b1065930bf350c2f). All sequences were compared to a database of reference rhodopsins to collect homologous sequences using MMseqs2 (68) and multiple alignments were built for each candidate sequence with mafft (–localpair) (69). These alignments were used as input to Polyphobius for the prediction of putative transmembrane helices (70). Only sequences with seven transmembrane helices and a lysine (K) residue in TM7 were retained.

### Gene context analysis.

Protein coding genes from all collected contigs harboring Alt-rhodopsins (*n* = 349) were predicted *de novo* by Prodigal v2.6.3 (66) in metagenomic mode (–p meta). Inferred protein sequences were annotated using a local installation of Interproscan (71) and by scanning them against the Protein Families (PFAM v.31) database with the Perl script pfam_scan.pl (available from ftp://ftp.ebi.ac.uk/pub/databases/Pfam/Tools). Annotation was also performed by scanning proteins with hmmsearch (67) against the COGs (clusters of orthologous groups) (72) and TIGRFAMs (73) HMM databases (E value ≤ 1e-3). BlastKOALA (74) was used to assign KO numbers to predicted orthologous proteins. To facilitate gene-context analysis for Alt-rhodopsins, the collection of contigs was clustered based on shared homologous proteins (i.e., requiring a minimum of 2 shared genes between any 2 members). For this purpose, all predicted proteins were clustered together using MMseqs2 (68) in easy-cluster mode (–cluster-mode 1 –c 0.5 –s 7.5) to identify homologs. Protein clustering information was further used to group contigs based on shared gene content. Contig clusters were plotted using Gcluster (75) with default parameters and showing consensus gene annotation generated as previously described. Manual curation of plotted clusters involved removal of contigs with less than 5 genes and collapsing of nearly identical ones while recording their number.

### Taxonomic classification of contigs.

Taxonomy was assigned to protein-coding genes we previously predicted within rhodopsin-encoding contigs by screening them with MMseqs2 (68) (“search” option, default parameters) against the annotated proteomes from the Genome Taxonomy Database (GTDB; release 95) (41). We followed a very conservative approach to pinpoint the taxonomic origin of these contigs, considering only those of at least 5 kb in length, and a minimum of 60% of genes giving best hits to the same phylum. Shorter contigs were retained as unclassified.

### Phylogenetic trees of rhodopsins.

The tree shown in Fig. 1 contains 2199 sequences of which 694 were reference rhodopsin sequences and the remaining 1505 were identified from our scan. These 1505 sequences were chosen as representatives of the total 6478 rhodopsin sequences identified in our scan. We chose to cluster all identified sequences at a 90% level of identity using MMseqs2 (easy-cluster) to include only representative ones in the tree. Additionally, all rhodopsin sequences >350 aa were excluded. Multiple alignments were performed using mafft (69) and a maximum likelihood tree was made using iqtree2 (76) with automatic model selection performed by ModelFinder (77), and 1000 iterations of ultrafast bootstrapping with 1000 rounds of SH-aLRT testing (-alrt 1000 -B 1000) (78).

The tree shown in Fig. 3 contains 834 sequences of which 226 were reference rhodopsins and 608 were identified from our scan. This reduced set of rhodopsins was obtained from the initial collection of 2201 sequences following clustering at 70% identity using MMseqs2. Sequences were aligned using PASTA (79) (default parameters) and tree construction performed by iqtree2 (v 2.1.2) with the same parameters used for the original tree. Phylogenetic tree pruning meant to highlight the Type-1/HeR split and manual annotations of the subtree were carried out in FigTree v 1.4.3 (https://github.com/rambaut/figtree/).

### Domain predictions and structural analyses.

Sequence-based domain predictions were carried out using Pfam (80), the Conserved Domain Database (81), HMMER (82), and HHPred (83). Structure prediction and domain definitions for selected sequences were performed using Alphafold2 (30) provided via ColabFold (84). Protein structures were visualized using ChimeraX (85). Structure-based searches were carried out using VAST (31). Transmembrane helix predictions were performed using Polyphobius (70) and supplemented wherever necessary with additional predictions from TOPCONS (86) and Phobius (87).

### Identification of NCLDV contigs.

Rhodopsin-encoding contigs of at least 5 kb (*n* = 485) were scanned with ViralRecall (26) to identify signatures of putative nucleocytoplasmic large DNA viruses (NCLDVs). The minimum number of viral hits to be reported by ViralRecall was reduced to 1 (-g 1) from the default value of 4 hits. We further classified contigs according to ViralRecall scores into four categories: NCLDV_high (≥5), NCLDV_medium (<5, ≥2), NCLDV_low (<2, >0), and non_NCLDV (<0) (results shown in Tables S1 and S2).

### Ancestral sequence reconstruction.

The evolutionary history of TM3 amino acid motifs from collected rhodopsins was inferred by ancestral sequence reconstruction (ASR) (88). In brief, the rhodopsin alignment generated for the reduced rhodopsin tree (*n* = 834 sequences; available at https://figshare.com/s/f2d7b1065930bf350c2f) was used as input in iqtree2 (v 2.1.2) with the –asr option specified and best model previously chosen according to the Bayesian information criterion (BIC) (–perturb 0.2 –nstop 500 -B 1000 -m LG+I+G4 –alrt 1000 -asr). Results were filtered to keep only ancestral sequence positions with a probability cutoff ≥0.4. TM3 motifs were identified in final ASR sequences by comparison to references and were indicated for 8 nodes in the phylogenetic tree shown in Fig. 3.

### Data availability.

All sequences used in this work, including reference sequences and derived data such as alignments and phylogenetic trees, have been deposited at FigShare at https://figshare.com/s/f2d7b1065930bf350c2f and are publicly available for download as of the date of publication.
